# The oral-brain axis: can periodontal pathogens trigger the onset and progression of Alzheimer’s disease?

**DOI:** 10.3389/fmicb.2024.1358179

**Published:** 2024-02-01

**Authors:** Ruohan Li, Junnan Wang, Wei Xiong, Yu Luo, Huixian Feng, Heng Zhou, Youjian Peng, Yan He, Qingsong Ye

**Affiliations:** ^1^Center of Regenerative Medicine, Renmin Hospital of Wuhan University, Wuhan University, Wuhan, China; ^2^Department of Stomatology, Renmin Hospital of Wuhan University, Wuhan University, Wuhan, China; ^3^Institute of Regenerative and Translational Medicine, Tianyou Hospital, Wuhan University of Science and Technology, Wuhan, China

**Keywords:** Alzheimer’s disease, periodontal pathogen, neuroinflammation, periodontitis, neurodegeneration

## Abstract

Alzheimer’s disease (AD) is the most prevalent form of dementia, characterized by a progressive cognitive decline. Sporadic AD, accounting for more than 95% of cases, may arise due to the influence of environmental factors. It was reported that periodontitis, a common oral ailment, shares several risk factors with AD, including advanced age, smoking, diabetes, and hypertension, among others. Periodontitis is an inflammatory disease triggered by dysbiosis of oral microorganisms, whereas Alzheimer’s disease is characterized by neuroinflammation. Many studies have indicated that chronic inflammation can instigate brain AD-related pathologies, including amyloid-β plaques, Tau protein hyperphosphorylation, neuroinflammation, and neurodegeneration. The potential involvement of periodontal pathogens and/or their virulence factors in the onset and progression of AD by the oral-brain axis has garnered significant attention among researchers with ongoing investigations. This review has updated the periodontal pathogens potentially associated with AD, elucidating their impact on the central nervous system, immune response, and related pathological processes in the brain to provide valuable insights for future research on the oral-brain axis.

## 1 Introduction

Cognitive impairment, characterized by the decline of memory, language, attention, executive function, and other cognitive domains, is a grave public health concern globally. The World Alzheimer’s Disease (AD) Report 2021 revealed that cognitive impairment affected more than 55 million individuals globally, predominantly in middle-aged and older age cohorts (Alzheimer's Disease International, 2021; Lu et al., 2023). Severe cognitive impairment presents as dementia, with AD being the most prevalent form. AD accounts for 60–80% of dementia cases and positions it as the fifth leading cause of mortality globally (Hodson, 2018). As the population ages, the incidence of AD is escalating. In 2015, 47 million individuals were diagnosed with AD globally, with projections indicating a surge to 75 million by 2030 and potentially to 132 million by 2050, posing a substantial social and economic burden (Taati Moghadam et al., 2022).

AD manifests as a progressive decline in cognitive function, accompanied by pathological alterations (Kitazawa et al., 2005). The characteristic pathological features of AD encompass the accumulation of amyloid-β (Aβ) peptide and neuronal fibrillary tangles (NFT) resulting from Tau hyperphosphorylation in the brain. Furthermore, AD patients exhibit neuroinflammation associated with infection, characterized by microglial activation and altered profiles of inflammatory factors (Park et al., 2018). According to pathogenic characteristics, AD is classified into two types: familial early-onset and sporadic late-onset. The former could be attributed to the mutations in the genes of presenilin 1, presenilin 2, and the Aβ precursor protein (AβPP), which cause AβPP cleavage, inducing extensive Aβ formation and deposition and early-onset cognitive impairment. The latter remains to be identified and may occur in the context of yet unidentified gene mutations, DNA oxidative damage, and other factors (Dorszewska et al., 2016). Currently, the inheritance of apolipoprotein ɛ4 (APOE ɛ4) gene is considered as the primary cause for sporadic AD, while environmental factors may play an indispensable role in modulating APOE ɛ4 expression (Taati Moghadam et al., 2022). Among patients with AD, sporadic late-onset AD accounts for over 95% of cases, suggesting a potential role of environmental factors in AD pathogenesis (Sansores-Espana D. et al., 2021; Panzarella et al., 2022).

Epidemiological studies have demonstrated that periodontitis is identified as an autonomous risk factor contributing to cognitive impairment (Ide et al., 2016; Iwasaki et al., 2019; Sung et al., 2019; Lu et al., 2023). The severity of periodontitis is significantly associated with cognitive dysfunction, as patients with periodontitis demonstrate a heightened propensity for cognitive impairment in comparison to individuals with healthy periodontal tissue (Sansores-Espana D. et al., 2021; Lu et al., 2023). Moreover, the severity of cognitive impairment in patients with severe periodontitis is three times higher compared to those with mild or no periodontitis, while elderly individuals with alveolar bone resorption face a 2.4-fold increased risk of cognitive dysfunction compared to those without such resorption (Gil-Montoya et al., 2015; Shin et al., 2016). Periodontitis is an inflammatory condition affecting the periodontal tissues, resulting from dysregulation of the oral microbiome. It shows progressive loss of periodontal structures, including gingivitis, periodontal ligament, and alveolar bone (Holmer et al., 2021). Severe periodontitis can even lead to tooth loss. According to previous studies, both AD and periodontitis are highly prevalent among middle-aged and older adults, sharing many similar risk factors, including age, smoking, diabetes, hypertension, and others (Jia et al., 2020). As the initiating factor of periodontitis, the oral microbiota is transmitted and extensively colonizes throughout the oral cavity (Schmidt et al., 2019). Periodontal pathogens are likely to play a pivotal role in the etiology and progression of cognitive impairment (Carter et al., 2017; Sczepanik et al., 2020).

Initial investigations discovered that the levels of serum IgG for bacteria linked to periodontitis, including *Porphyromonas gingivalis* (*Pg*), *Tannerella forsythia*, *Aggregatibacter actinomycetemcomitans* (*Aa*), *Treponema denticola* (*Td*), and *Campylobacter rectus*, were correlated with the likelihood of AD (Noble et al., 2014). Moreover, the findings from oral microbiome research have demonstrated that the subgingival microbiota in individuals with cognitive impairment exhibited characteristic alterations akin to periodontal disease (Holmer et al., 2021). Additional studies revealed that *Pg*, *Fusobacterium nucleatum* (*Fn*), *Prevotella intermedia*, and *Veillonella parvula* were significantly elevated in AD patients (Taati Moghadam et al., 2022; Guo et al., 2023). It suggests a plausible association between periodontal pathogens and AD to a certain extent (Taati Moghadam et al., 2022; Guo et al., 2023).

To date, the precise role of periodontal pathogens in AD pathology remains elusive. The concept of the oral-brain axis and its potential role in AD pathogenesis has garnered significant attention. Periodontal pathogens and/or their toxins could potentially trigger AD-related pathological changes and cognitive decline through the oral-brain axis. From the perspective of oral microbial infection, this review examines the periodontal pathogens and/or their toxins significantly associated with AD. It delineates the mechanisms and pathways of how these pathogens potentially affect the immune response and AD related pathological processes in the central nervous system (CNS), thereby offering valuable insights for future research.

## 2 Periodontal pathogens potentially associated with AD

### 2.1 Porphyromonas gingivalis

*Pg* is among the most prevalent periodontal pathogens and has been extensively studied, with a strong correlation established between *Pg* and AD incidence and progression. Initial clinical investigations revealed significantly elevated serum *Pg* IgG levels in subjects with cognitive impairment, which exhibited a robust correlation with the severity of periodontal disease (Noble et al., 2009). Carter et al. (2017) conducted a genome-wide association study utilizing GWASdb. They discovered that host genes interacting with *Pg* were significantly enriched in those associated with AD. In addition, mice or rats chronically infected with *Pg* orally exhibit *Pg* colonization in the hippocampal and cortical layers, accompanied by classic AD-like pathology such as neuroinflammation, neurodegeneration, Tau phosphorylation, and the formation of both intracellular and extracellular Aβ plaques and NFT (Wu et al., 2017; Ilievski et al., 2018; Dominy et al., 2019; Díaz-Zúñiga et al., 2020; Haditsch et al., 2020; Huang et al., 2021; Sansores-España L. D. et al., 2021; Tang et al., 2021; Zeng et al., 2021; Hao et al., 2022).

*Pg* has the ability to invade host cells within cytoplasmic or lysosomal-like structures as autophagic vacuoles or multivesicular bodies (Haditsch et al., 2020). As a result, *Pg* is likely to infect neurons and elicit toxic effects. The primary virulence factors of *Pg* include gingipain, lipopolysaccharide (LPS), bacterial metabolites, and outer membrane vesicles (OMVs) encapsulating a repertoire of key virulence factors, including LPS, gingipain, and other enzymes. Relevant studies have demonstrated the detection of *Pg* DNA and associated virulence factors in AD patients’ brains, and *Pg*-derived gingipain, LPS, and OMVs have been shown to induce AD-like pathology in wild-type mice (Wu et al., 2017; Dominy et al., 2019; Wei et al., 2020; Gong et al., 2022). The potential signaling pathways of periodontal pathogens and/or their virulence factors affecting AD-related pathology via the oral-brain axis were shown in Table 1.

**Table 1:** The potential signaling pathways of periodontal pathogens and/or their virulence factors affecting AD-related pathology via the oral-brain axis.

Pathogens/virulence factors	Potential signal pathways	References
*Pg*	Enhancing the accumulation of Aβ in peripheral monocytes/macrophages through activation of CatB/NF-κB signaling;	Nie et al. (2019)	Increasing the transport of Aβ from the periphery into the brain through up-regulation of receptors for advanced glycation end products expression in brain endothelial cells;	Zeng et al. (2021)	Exhibiting Tau hyperphosphorylation, up-regulating IL-1β, and decreasing PP2A in the hippocampus of rats, and promoting PP2A expression mitigated Tau hyperphosphorylation in HT22;	Tang et al. (2021)	Inducing C1q overexpression to amplify microglial phagocytosis, resulting in neuroinflammation and synapse loss. Furthermore, blocking C1q reduced the effect of synaptic loss.	Hao et al. (2022)
*Pg*-derived LPS	Activating microglia through the TLR2/TLR4-mediated NF-κB/STAT3 signaling pathway, upregulating the inflammatory factors expression, including IL-1β, TNF-α, IL-6, IL-23, and IL-17A, leading to cognitive impairment in mice;	Kirkley et al. (2017), Qiu et al. (2021), Zhang et al. (2021)	Activating TLR/NF-κB signaling to benefit CatB-mediated Aβ accumulation in the neurons;	Wu et al. (2017), Zhang J. et al. (2018)	Triggering the release of inflammatory factors from microglia in the brain mediated by GSK-3β in the mouse models.	Jiang et al. (2021)
*Pg*-derived OMVs	Activating the NLRP3 inflammasome, leading to the induction of IL-1β, TNF-α, and NF-κB production and contributing to Tau phosphorylation and neuronal degeneration;	Wei et al. (2020), Gong et al. (2022)	Triggering the release of inflammatory factors from microglia in the brain by activating GSK-3β.	Wei et al. (2020), Jiang et al. (2021)
*Td*	Up-regulating the expression of BACE1 and presenilin 1, promoting the accumulation of Aβ_1-42_ and Aβ_1-40_ in the hippocampus of mice;	Su et al. (2021)	Enhancing Tau hyperphosphorylation at Ser396, Thr181, and Thr231 residues through upregulating GSK-3β kinase activity in mice and was verified *in vitro* study with BV2 and N2a cells;	Tang et al. (2022)	Decreasing BCL-W and increasing the second mitochondria-derived activator of caspases by activating the MAPK/JNK pathway to neuronal apoptosis in the hippocampus of the mice.	Wu L. et al. (2022)
*Fn*	Activating microglia, enhancing the expression of TNF-α and IL-1β *in vitro*, and resulted in cognitive impairment, Aβ deposition and Tau hyperphosphorylation in the mouse cerebrum.	Wu H. et al. (2022)
*Aa*-derived serum type b-LPS	Resulting in the release of microglia-mediated inflammatory factors, neuroinflammation, and Aβ production in the hippocampal cells *in vitro*.	Díaz-Zúñiga et al. (2019)
*Aa*-derived OMVs	Stimulating TNF-α and IL-6 production in the cerebral cortex through TLR-8 and NF-κB pathways to affect the brain immunity.	Han et al. (2019); Ha et al. (2020)

### 2.2 Treponema denticola

*Td*, the predominant spirochete, is situated within the gingival and subgingival plaque, playing a pivotal role in the progression of chronic periodontitis. The involvement of spirochetes in AD pathology was initially proposed by Miklossy (1993). After summarizing previous studies, she discovered that the spirochete detection rate in the brain tissue of patients with AD surpassed 91.1% (451 of 495), with a significantly increased level of multiple treponemal species compared to the control group (Miklossy, 2011). Subsequent population-based studies reached similar conclusions, and *Td* was substantially higher in brain samples from patients with AD (14 out of 16) compared to healthy controls (4 out of 18) (Poole et al., 2013). It suggests a potential association between spirochetes and cognitive impairment.

Previous studies have found that spirochetes invading the brain may form intracellular and extracellular biofilms involved in Aβ deposition (Allen, 2021). In the speculative pathway, spirochetes within neurons foster Aβ production while producing biofilm and initiate subsequent events associated with AD; extracellular biofilms of spirochetes may activate immune responses that facilitate Aβ deposition (Miklossy, 2011). In addition, the Aβ-coated spirocholitic biofilms in the extracellular senile plaques may cause the production of NF-κB and TNF-α by attaching Toll-like receptor (TLR) 2, thereby promoting Aβ deposition (Allen, 2016). Subsequent research revealed that *Td* induced Aβ accumulation, Tau hyperphosphorylation, neuroinflammation, and neuronal apoptosis in the hippocampus (Su et al., 2021; Tang et al., 2022; Wu L. et al., 2022). Preliminary studies confirmed that orally infected *Td* may up-regulate the expression of AβPP cleaving enzyme 1 (BACE1) and Presenilin 1, thereby promoting the accumulation of Aβ_1-42_ and Aβ_1-40_ in the hippocampus of mice (Su et al., 2021). Subsequently, Tang et al. (2022) discovered that *Td* infection also elicited Tau hyperphosphorylation and neuroinflammation in the hippocampus of mice. Specifically, oral administration of *Td* led to enhanced phosphorylation of Tau at Ser396, Thr181, and Thr231 residues through upregulation of GSK3β kinase activity. Moreover, *Td* played a significant role in promoting neuronal apoptosis, and it was likely to decrease BCL-W expression and increase the second mitochondria-derived activator of caspases by activating the MAPK/JNK pathway, thereby facilitating neuronal apoptosis (Wu L. et al., 2022).

### 2.3 Fusobacterium nucleatum

Another periodontal pathogen associated with AD is *Fn*. *Fn* is a Gram-negative anaerobe functioning as a copolymerizing bridging organism. The virulence mechanisms of *Fn* involve colonization, invasion, induction of an abnormal inflammatory response, and immune evasion. *Fn* is known to possess virulence factors, including FadA, Fap2, and LPS. Initial findings demonstrated a significantly higher concentration of antibodies to *Fn* in the serum of patients with AD compared to healthy individuals (Sparks Stein et al., 2012). Furthermore, the load of *Fn* in oral microorganisms was significantly higher in patients with AD compared to the control group (Panzarella et al., 2022; Taati Moghadam et al., 2022). Chronic oral administration of *Fn* led to spatial learning impairment, neurodegeneration, Aβ accumulation, and Tau hyperphosphorylation in mice, accompanied by an increased LPS load in the serum (Yan et al., 2022). *Fn* could also activate microglia and inflammatory pathways *in vitro* experiments (Wu H. et al., 2022). In addition, by establishing animal models of oral infection, *Fn* was found to cause alveolar bone resorption and promote inflammatory responses in the brain, which aggravated Aβ deposition and increased Tau phosphorylation in 5xFAD mice (Wu H. et al., 2022).

### 2.4 Aggregatibacter actinomycetemcomitans

*Aa* is the most aggressive periodontal pathogen, characterized by distinct serotypes and virulence based on LPS antigenicity. Treatment with *Aa*-derived serum type b-LPS resulted in microglia-mediated inflammatory factor release, neuroinflammation, and Aβ production in hippocampal cells (Díaz-Zúñiga et al., 2019). Additionally, *Aa*-derived OMVs traversing the peripheral circulation can reach brain tissue, stimulating the release of inflammatory factors, including TNF-α and IL-6 (Han et al., 2019; Ha et al., 2020). The extracellular RNA within OMVs may contribute to brain immunity. Although the link between *Aa* and AD remains tenuous, *Aa* is likely involved in AD progression, and further studies *in vivo* are needed.

### 2.5 Other periodontal pathogens

Other periodontal pathogens may be associated with AD. Noble et al. (2014) proposed that serum IgG levels of bacteria linked to periodontitis, including *Tannerella forsythia* and *Campylobacter rectus*, may be associated with AD. Furthermore, *Prevotella intermedia* and *Veillonella parvula levels* increased in AD patients’ gingival crevicular fluid samples (Taati Moghadam et al., 2022; Guo et al., 2023). Notably, *Filifactor alocis* can synergize with other pathogens to impair gingival epithelium’s immune and preventive capabilities. It may enhance the invasive ability of *Pg*, *Fn*, and *Prevotella intermedia* through vesicle-mediated internalization while exhibiting good abilities to produce ammonia to protect other pathogens. Therefore, *Filifactor alocis* may be crucial in mediating bacterial migration from oral tissue to distant tissues, which could indirectly contribute to AD-related pathologies (Aruni et al., 2014, 2015). However, more substantial evidence is required to verify their potential involvement in AD pathology.

## 3 The virulence factors of periodontal pathogens affecting AD

Periodontal pathogens possess the capacity for extensive invasion, and microbial colonization is a vital mechanism for their pathogenic function. Periodontitis pathogenic bacteria can migrate from gingival tissue or deep periodontal pockets to distant tissues and organs, including the brain parenchyma, instigating AD-related pathological changes (Poole et al., 2013; Chukkapalli et al., 2016; Dominy et al., 2019; Qian et al., 2021; Aravindraja et al., 2022; Lei et al., 2023). The virulence of periodontal pathogens primarily relies on various virulence factors, which facilitate AD-like pathology (Poole et al., 2013; Chukkapalli et al., 2016; Dominy et al., 2019; Lei et al., 2023).

### 3.1 Gingipain

The accumulating evidence underscores the critical role of gingipains in AD pathology. Gingipains, a class of cysteine proteases comprising lysine-specific gingipains (Kgp) and arginine-specific gingipains known as RgpA and RgpB, are synthesized as soluble entities or OMVs and transported from the periplasmic transmembrane layer to the extracellular space through the type IX secretion system (Nakayama, 2015; Benedyk et al., 2019; Lei et al., 2023). Dominy et al. (2019) reported that gingipains were present in over 90% of AD brains, and administration of small-molecule inhibitors of gingipains significantly diminished *Pg* load and Aβ_1-42_ production in the mouse brain, as well as arrested neurodegeneration. In wild-type mice orally administered *Pg*, gingipains were detected in the intranuclear, perinuclear, and extracellular regions of neurons, microglia, and astrocytes in the hippocampus, concurrent with neurodegeneration, Aβ deposition, and neuroinflammation (Liu et al., 2017; Haditsch et al., 2020). Gingipains also forestalled *Pg*-containing autophagosomes from binding to lysosomes, enabling bacteria to survive in autophagosomes and elicit toxic effects (Yamatake et al., 2007).

### 3.2 LPS

LPS, the predominant cell wall constituent in Gram-negative bacteria, plays a pivotal role in virulence. Its structural components encompass lipid A, core oligosaccharides, and the O-antigen. Lipid A serves as the primary bioactive moiety within LPS by engaging marrow differentiation protein 2 (MD2) and inducing TLR4 dimerization, initiating downstream signaling cascades and promoting proinflammatory factor secretion (Qiu et al., 2021). LPS persistently stimulates host immune cells, including monocytes/macrophages, and induces the release of diverse bioactive substances, such as cellular inflammatory factors, reactive oxygen species, nitric oxide, and lysosomal enzymes, among others (Jain and Darveau, 2010). It leads to inflammation reactions, cellular damage, and/or apoptosis. Furthermore, LPS contains diverse lipid A species that may activate innate immune responses through TLR2 and TLR4 (Darveau et al., 2004). Inflammatory stimulation can induce neuronal apoptosis and cognitive dysfunction. Chronic exposure to *Pg*-derived LPS leads to synaptic loss and memory impairment in mice, as well as Aβ deposition (Gu et al., 2020). Moreover, *Pg*-derived LPS may initiate neuronal inflammation via the TLR4/NFkB pathway and induce intracellular Aβ accumulation in neurons, dependent on cathepsin B (CatB) (Wu et al., 2017; Zhang J. et al., 2018; Nie et al., 2019; Zeng et al., 2021).

### 3.3 OMVs

Notably, the influence of periodontal pathogens on AD extends beyond mere microbial colonization. The DNA of *Td* and *Fn* was absent from the brain tissue of mice following oral infection for several weeks (Foschi et al., 2006; Wu H. et al., 2022; Yan et al., 2022). It suggests that the remote action of bacteria-associated toxins also plays a significant role. The role of bacterial OMVs has garnered considerable attention. OMVs, secreted by Gram-negative bacteria with a diameter of approximately 50 to 250 nm and a double-layer spherical membrane-like structure, have demonstrated the ability to traverse the blood–brain barrier (BBB) and access the brain following cardiac administration. In addition, caudal-intravenously administered OMVs were found to be delivered sequentially to meningeal macrophages and cortical microglia (Han et al., 2019; Ha et al., 2020). *Pg*-derived OMVs are trim and adhesive, making them more stable than strains and less susceptible to host-derived proteases. It enables them to penetrate deep tissues more effectively and activate host inflammatory responses (Das et al., 2019). *Pg*-derived OMVs can trigger inflammatory responses, Tau phosphorylation, and neuronal degeneration (Wei et al., 2020; Gong et al., 2022). The toxicity of OMVs depends on lipids, proteins, and nucleic acids. Within the biological structure of OMVs, LPS situates on the surface of the cell wall, and gingipains localize on the cell wall (Farrugia et al., 2020). OMVs-derived LPS and gingipains also function as toxin factors, similar to bacteria. Moreover, bacterial extracellular DNA has been observed to induce the formation of misfolded Tau aggregates and Aβ aggregation, indicating a potential role for OMVs-derived extracellular DNA in AD (Tetz et al., 2020; Tetz and Tetz, 2021). OMVs may act as carriers of microbial toxin factors, initiating chronic inflammatory activation and tissue damage to remote tissues, including brain tissue, via their sustained targeting effects with nanoscale biological structures.

### 3.4 Others

Novel sphingolipids, namely glycerol phosphate dihydroceramide (PGDHC) and ethanolamine phosphate dihydroceramide (PEDHC), are lipid-derived virulence factors of bacteria (Singhrao et al., 2015). The interaction between PEDHC and TLR2/TLR4 initiates a robust inflammatory response in young mice (Shaik-Dasthagirisaheb et al., 2015). Moreover, PGDHC ceramides, derived from *Pg*, have been shown to enhance Aβ deposition, Tau hyperphosphorylation, and the production of senescence-associated secretory phenotype (SASP) factors such as β-galactosidase, CatB, TNF-α, and IL-6 (Yamada et al., 2020). Consequently, PGDHC may represent a novel class of bacterial virulence factors associated with periodontitis-associated AD.

In experimental rodent models of periodontitis, capsular-bearing *Pg* strains exhibit greater virulence than capsular-defective mutants, leading to augmented immune responses, osteoclast activity, and alveolar bone resorption (Vernal et al., 2014a,b). Microglia, the resident macrophages of the central nervous system, can differentiate between various bacterial antigens, triggering distinct pathways based on the recognized antigen (Zhang Y. et al., 2018; Haditsch et al., 2020). *Pg* capsular serotypes might contribute to the activation of microglia and AD pathogenesis. Prior studies suggest that more virulent encapsulated *Pg* strains could instigate brain Aβ deposition, Tau phosphorylation, neuroinflammation, and cognitive decline in rats (Díaz-Zúñiga et al., 2020). As bacterial virulence factors, composed of capsular polysaccharides, possess a higher capacity to activate brain inflammatory responses, they might be crucial in stimulating innate immunity and *Pg*-induced AD-like pathology.

## 4 The pathways of periodontal pathogens affecting CNS

**Figure 1:**
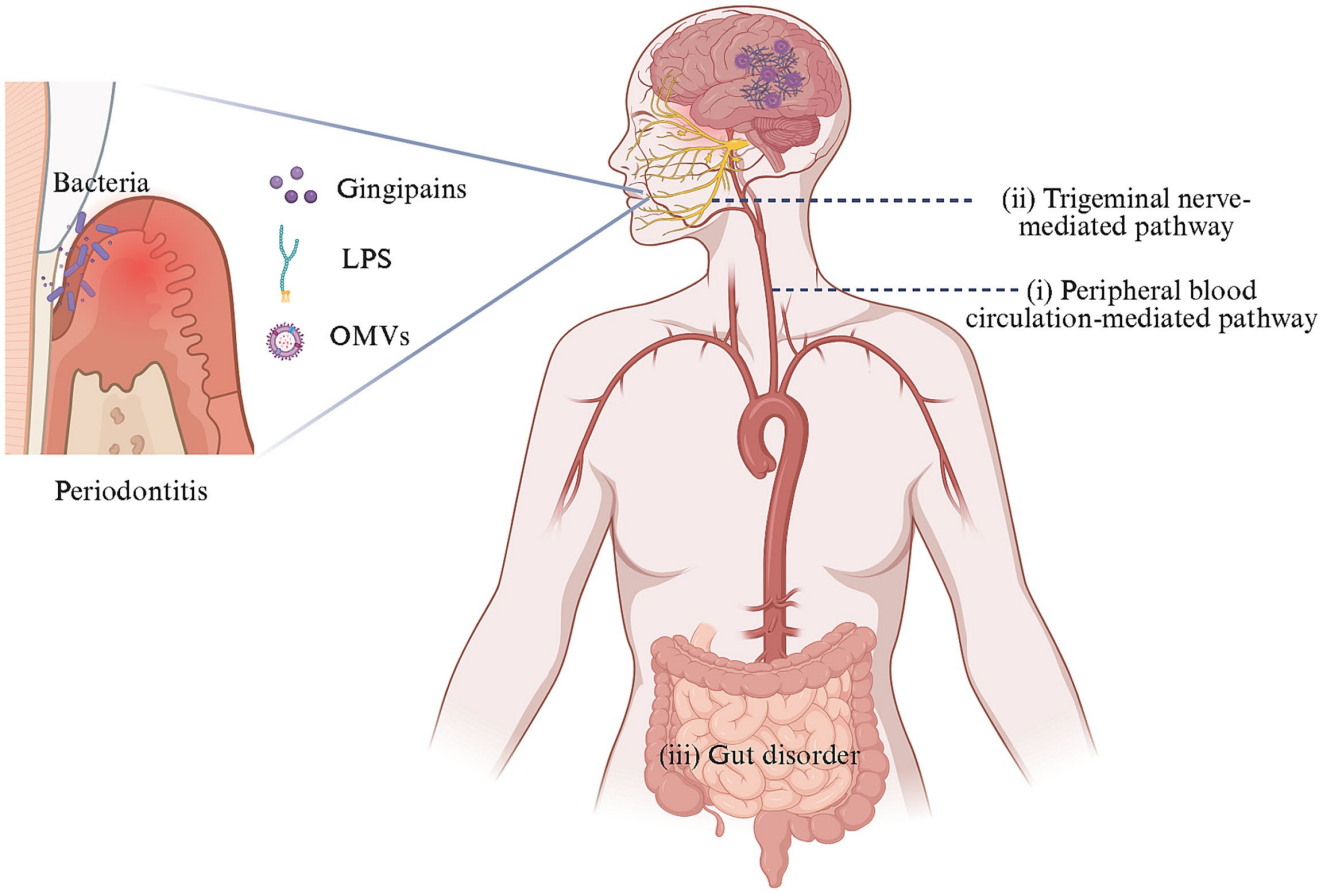
Oral-brain axis-related pathways of AD caused by periodontal pathogens. In the context of periodontitis, dysregulated oral microbes could invade and colonize the brain directly through two mechanisms: (i) crossing the BBB via peripheral blood circulation and (ii) reaching the brain through trigeminal neuron-mediated trans neural pathway. Additionally, these pathogens might indirectly impact the brain by perturbing gut microbiota. These processes contribute to the occurrence and progression of AD-like pathology in the brain. Created with BioRender.com.

The entry of periodontal pathogens into brain tissue and their role remains elusive, currently posing as a research hotspot. A multi-pathway oral-brain axis mechanism has been proposed (Figure 1).

(1) The transmission and diffusion of periodontal pathogens in the peripheral circulation system is a leading research direction. Periodontal pathogens may access the brain through three potential peripheral circulation pathways: (a) Oral microorganisms and/or their toxins could damage the mucosal barrier, diffuse into the bloodstream, and enter the brain by disrupting the BBB (Farrugia et al., 2020; Sansores-España L. D. et al., 2021; Nonaka et al., 2022; Pritchard et al., 2022; Guan et al., 2023; Lei et al., 2023); (b) The periventricular organs lack a continuous BBB, allowing periodontal pathogens and/or their toxins to enter the brain through this route potentially (Olsen and Singhrao, 2015); (c) Bacteria can cause systemic inflammation and stimulate the release of inflammatory factors that activate corresponding receptors on brain capillary endothelial cells or perivascular macrophages and may enter the CNS through the glymphatic pathway, given that after traversing the parenchymal capillaries of the brain, bacteria are likely to migrate toward the meninges via the glymphatic pathway (Chou et al., 2016; Coureuil et al., 2017).
(2) The neural migration pathway of periodontal pathogens has garnered increasing attention. Bacteria and/or their toxins could migrate to the trigeminal ganglion along the peripheral end of craniofacial nerves, such as the olfactory trigeminal nerve, and retrograde into the brain (Riviere et al., 2002; Su et al., 2021; Ma et al., 2023).
(3) Periodontal pathogens might cause cognitive impairment by disrupting changes in the gut microbiota (Chi et al., 2021; Yan et al., 2022).

BBB functions as the primary defense line between the circulation system and CNS, effectively preventing the infiltration of peripheral bacteria and/or their virulence factors into the brain (Gong et al., 2023). Comprised primarily of brain microvascular endothelial cells (BMECs), astrocytes, pericytes, and a basement membrane, it safeguards cerebral integrity (Nation et al., 2019). However, factors such as bacterial and/or toxin-induced vascular inflammation, cerebral vessel aging, and increased blood inflammatory mediators in the blood can disrupt the BBB and its permeability, allowing bacteria and/or their toxins to access the brain parenchyma. Studies have discovered that periodontal pathogens and/or their toxins can increase BBB permeability (Farrugia et al., 2020; Nonaka et al., 2022; Pritchard et al., 2022; Sato et al., 2022; Lei et al., 2023). BBB disruption is characterized by an excessive uptake of BMECs endocytosis (transcellular pathway) and a loss of BMECs cell-to-cell connectivity (extracellular pathway) (Nation et al., 2019). *In vitro* and *in vivo* research, *Pg*, *Pg*-derived OMVs, and LPS have been shown to impair BBB integrity, thereby increasing its permeability (Nonaka et al., 2022; Pritchard et al., 2022; Lei et al., 2023). *Pg* may enhance BBB permeability through the Mfsd2a/Cav-1-mediated transcellular transport pathway, while *Pg*-derived LPS can induce the expression of IL-6 and CCL2 in hCMEC/D3 cells through the TLR4/NF-κB pathway, leading to endothelial inflammation and BBB disruption (Sato et al., 2022). Notably, gingipains appear to play a substantial role in BBB damage. Peripheral *Pg* and *Pg*-derived OMVs can deliver gingipain to BMECs to disrupt the BBB (Nonaka et al., 2022; Lei et al., 2023). Furthermore, gingipain may mediate BBB dysfunction through Rgp-Cav-1 interactions or affect the proteolysis of endothelial-cell adhesion proteins such as CD31 (Farrugia et al., 2020; Lei et al., 2023).

In addition to blood circulation, the mode of trans nerve migration of periodontal pathogens has garnered researchers’ attention. A study discovered Aβ deposition in wild-type mice following oral infection with *Td* and found 16S rDNA derived from *Td* in a limited number of trigeminal ganglion samples (Su et al., 2021). Ma et al. (2023) later found that fluorescein-5-isothiocyanate-labeled OMVs could be seen in both the trigeminal ganglion and hippocampus regions following chronic exposure of the gingiva to *Pg*-derived OMVs, indicating the potential for trans-nerve migration of periodontal pathogenic OMVs to the brain.

## 5 Effects of periodontal pathogens on immunity response and neuroinflammation in the brain

The prevailing perspective posits that chronic inflammation plays a substantial role in the pathogenesis of AD. Inflammation could potentially drive the pathologies linked to Aβ deposition and Tau hyperphosphorylation in brain tissue, with alterations in microbiota serving as one of the crucial factors exacerbating chronic inflammation (Tang et al., 2021; Yan et al., 2022). It is widely accepted that low-level bacterial infections instigate systemic inflammation and subsequent detrimental events. Inflammation may initially arise from oral infections, which harbor a complex ecosystem comprising diverse microflora encompassing bacteria, fungi, and viruses. Moreover, periodontal pathogens and/or their toxins in the CNS could activate immune responses, leading to a cascade of interconnected events, resulting in neuroinflammation and cognitive impairment. The susceptibility to microbial infections is exacerbated with aging due to immuno-senescence. Consequently, the risk of oral inflammation markedly increases for middle-aged and older individuals, potentially accounting for the alterations in the oral microbiome (Yang et al., 2021).

Periodontal pathogens trigger heightened CNS inflammatory mediators and instigate neuroinflammatory responses. Firstly, these pathogens or their toxins can prompt peripheral local or systemic inflammatory responses, with the produced inflammatory mediators (such as IL-1β, TNF-α, and PGE2). These mediators have the potential to cross the BBB via systemic circulation and initiate neuroinflammation (Italiani et al., 2018; Tang et al., 2021; Hao et al., 2022; Wang et al., 2023). Secondly, the infiltration of periodontal pathogens or their toxins into brain tissue may induce microglial activation and subsequently trigger the release of inflammatory mediators. Research indicated a significant elevation of TNF-α and IL-1β levels in the brain tissues of patients with AD (Saffari et al., 2020; Wu H. et al., 2022). Neuroinflammation has also been observed in the hippocampus of mice and rats orally infected with *Pg*, concomitant with elevated TNF-α, IL-1β, and IL-6 (Ding et al., 2018; Ilievski et al., 2018; Díaz-Zúñiga et al., 2020; Tang et al., 2021). Additionally, most inflammatory markers in CSF augment in individuals with cognitive impairment, encompassing Oncostatin-M, endopeptidases (MMP-10, MMP-9, TIMP-4, etc.), and chemokine (CCL3) (Whelan et al., 2019; Yang et al., 2021). These inflammatory markers facilitate proinflammatory effects in the CNS and contribute to neuroimmune processes, resulting in neuronal damage and subsequent AD events (Bagheri-Mohammadi, 2021).

Precisely, microglia perform a vital function in instigating and governing neuroinflammation. Neuroinflammation is widely acknowledged as a significant contributory factor in the AD pathogenesis. As indigenous monocytes/macrophages within the CNS, microglia establish an immune surveillance system in the brain to regulate neuroinflammation. These cells are within the brain parenchyma and encompass M1 and M2 phenotypes (Bagheri-Mohammadi, 2021). Inflammatory mediators, such as LPS, Aβ, and I*FN*-γ, induce the activation of the M1 phenotype, whereas the M2 phenotype confers advantages in terms of inflammation reduction and tissue repair. Under normal physiological conditions, microglia preserve the regular functions of neurons and astrocytes by expressing soluble molecules to clear cell debris, polymerizing proteins, and executing synaptic pruning. Nevertheless, when stimulated by persistent inflammation, activated microglia can disseminate a multitude of humoral factors (including cytokines, chemokines, etc.) through autocrine or paracrine pathways, encompassing IL-1β, IL-6, and iNOS, expediting neuroinflammation and neurotoxic responses, leading to neuronal damage and Aβ deposition within the brain (Salter and Beggs, 2014; Kirkley et al., 2017; Tran et al., 2021; Wu H. et al., 2022).

**Figure 2:**
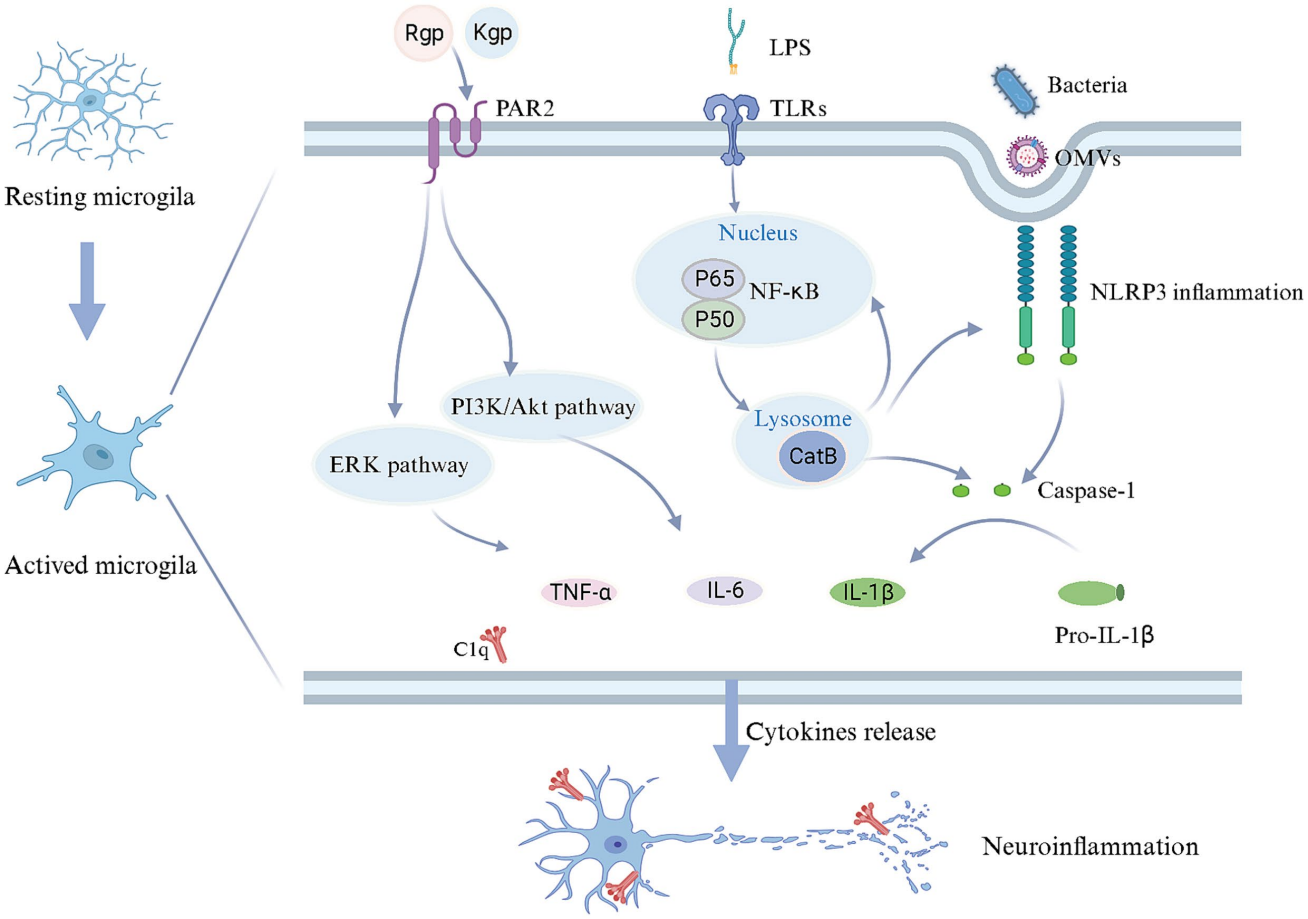
Mechanisms in microglial activation and neuroinflammation triggered by periodontal pathogens. Upon reaching the brain, periodontal pathogens and/or their toxins induce a transition of microglia from a quiescent state to an activated state. LPS can bind to TLR receptors on the cell membrane, starting CatB to facilitate IL-1β production through the NF-κB signaling pathway. Periodontal pathogens and OMVs may enter microglia via endocytosis, stimulating the caspase-1 production and IL-1β release through activating NLRP3 inflammasome, while CatB is also involved in NLRP3 inflammasome activation. Furthermore, gingipains derived from periodontal pathogens, upon binding to PAR2, could potentially contribute to releasing IL-6 and TNF-α through the PI3K/AKT and ERK pathways. As a result, microglia release an array of inflammatory mediators and complement C1q, inducing neuroinflammation. Created with BioRender.com.

Periodontal pathogens are implicated in the microglia activation and neuroinflammatory responses (Figure 2). Related studies have demonstrated that *Pg* and *Fn* stimulate microglial activation (Dominy et al., 2019; Wu H. et al., 2022). Upon *Pg* invasion of the brain, it elicits the secretion of UDP by microglia at the site of infection, triggering the elongation of microglial processes and transformation into an activated state (Takayama et al., 2016). TLRs, as the type I transmembrane proteins, play a pivotal role in the innate immune system by recognizing pathogen-derived macromolecules. *Pg*-derived LPS can activate microglia through the TLR2/TLR4-mediated NF-κB/STAT3 signaling pathway, upregulating the inflammatory factors expression, including IL-1β, TNF-α, IL-6, IL-23, and IL-17A, initiating an inflammatory cascade (Kirkley et al., 2017; Qiu et al., 2021; Zhang et al., 2021). *Pg* can also stimulate microglia through gingipains-mediated protease-activated receptor-2 activation. The subsequent activation of PI3K/Ark and ERK pathways could stimulate microglial activation, contributing to cell migration and inflammation (Liu et al., 2017). However, OMVs facilitate cellular communication and microglial activation. OMVs derived from *Pg* promote microglial activation and induce the secretion of pro-inflammatory cytokines IL-1β, TNF-α, and NF-κB (Wei et al., 2020; Gong et al., 2022). The extracellular RNA cargo carried by *Aa*-derived OMVs stimulates the TNF-α and IL-6 production in the cerebral cortex through TLR-8 and NF-κB pathways to affect the brain immunity (Han et al., 2019; Ha et al., 2020).

The activation of the inflammasome in microglia should not be disregarded, given its pivotal role in the intracellular innate immune system. Inflammasomes are multiprotein complexes that become activated during infection-induced neuroinflammation. Prior studies have highlighted the inflammasome activation in the AD brain, including NLRP1 and NLRP3, suggesting that *Pg* may contribute to this process (Olsen and Yilmaz, 2016; Saresella et al., 2016). The inflammasome senses cell integrity and participates in the homeostasis between the microbiota and the host. NLRP3 is particularly notable for its role in microbial pathogenesis. The activation of NLRP3 in microglia could drive Aβ deposition (Dominy et al., 2019; Sczepanik et al., 2020). Moreover, activated Inflammasome triggers the maturation of proinflammatory cytokines, leading to cellular pyroptosis while eliminating the intracellular niche for bacterial proliferation (Fleetwood et al., 2017; Dominy et al., 2019). Notably, *Pg*-derived OMVs have been shown to activate the NLRP3 inflammasome, leading to the induction of IL-1β production and contributing to Tau phosphorylation and neuronal degeneration (Gong et al., 2022).

The overactivation of the cerebral complement system has been implicated in the pathogenesis of AD (Hong et al., 2016; Hao et al., 2022). Inhibition or a deficiency of complement components may potentially alleviate AD-related pathology (Fonseca et al., 2004). Complement C1q in the brain is primarily derived from microglia (Fonseca et al., 2017; Hao et al., 2022). Additionally, activated complement components are abundant in the periodontal tissues with periodontitis. Consequently, in the context of periodontal infection, dysregulation of the complement system is most likely the driving force behind neuroinflammation observed in AD patients. It has been postulated that *Pg* induced C1q overexpression to amplify microglial phagocytosis, resulting in neuroinflammation and synapse loss. Furthermore, blocking C1q reduced the effect of synaptic loss (Hao et al., 2022). Therefore, the activation of Complement C1q may be crucial in exacerbating microglia activation.

Periodontal pathogens are also implicated in host adaptive immunity. CD4+ T cells are capable of secreting various cytokines and activating innate and adaptive immunity. Both T-helper (Th) and Treg cells actively contribute to the neuroinflammation. Th17 cells exhibit a pro-inflammatory effect and can release IL-17A, exacerbating neuronal apoptosis (Cho et al., 2019). Treg cells counteract the inflammatory effects of Th17 cells and preserve immune homeostasis (Salminen et al., 2020). *Pg*-derived LPS has been demonstrated to promote Th17 cell differentiation *in vitro* (Lewis et al., 2015; Zhang et al., 2019). Zhang et al. (2021) found that gingival injection of *Pg*-LPS triggers Th17/Treg imbalance by activating the STAT3 signaling pathway, leading to cognitive impairment in mice. Among the observed changes, there was an upregulation in the expression of Th17-associated cytokines (IL-1β, IL-17A, IL-21, and IL-22). At the same time, a downregulation was noted in Treg-related cytokines (IL-2 and IL-10) both in peripheral blood and brain tissues. This shift resembles the immune imbalance observed in brain tissue of AD patients (Oberstein et al., 2018).

## 6 The mechanisms of periodontal pathogens affecting AD

### 6.1 Mechanisms associated with Aβ deposition by periodontal pathogens

The short peptide structure Aβ is generated by the degradation of AβPP. In the presence of presenilin, AβPP initially undergoes hydrolysis by β-secretase 1 into β-N-terminal fragments and β-C-terminal fragments, followed by hydrolysis by γ-secretase. During this process, the dysregulation of BACE1, Presenilin 1, and Presenilin 2 leads to perturbations in AβPP degradation and subsequent Aβ production. Throughout the pathological progression of AD, Aβ deposition occurs both within and outside cells. Intracellular Aβ deposition is evident in the early stages of AD, while extracellular soluble Aβ oligomers induce cytotoxicity, potentially leading to the synaptic structure and abnormal function, initiating a cascade related to AD pathology (Huang et al., 2013; Su et al., 2021).

**Figure 3:**
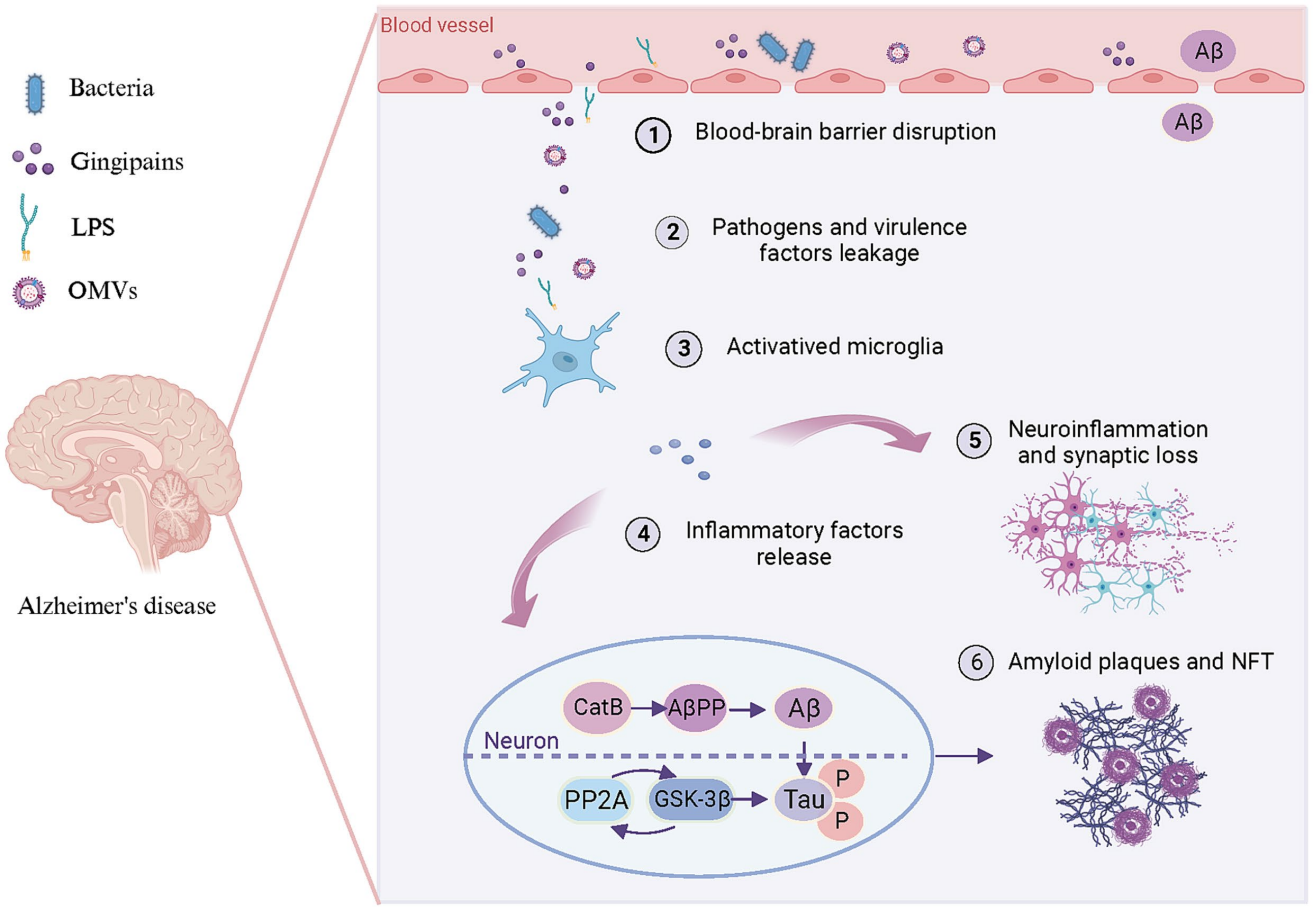
Pattern diagram illustrating the involvement of periodontal pathogens in brain lesions resembling AD. Periodontal pathogens and/or their toxins (1) disrupt the BBB, (2) lead to bacteria and virulence factors leakage into the brain. Subsequently, bacteria and virulence factors further (3) activate microglia and (4) lead to the release of inflammatory factors. These inflammation factors not only (5) trigger neuroinflammation and synaptic loss but (6) promote Aβ production and Tau hyperphosphorylation in neurons. On the one hand, pro-inflammatory factors can enhance CatB activity to participate in AβPP processing, thereby promoting Aβ generation and deposition of amyloid plaques. On the other hand, they can activate PP2A along with its downstream molecule GSK-3β to promote Tau hyperphosphorylation and subsequent formation of NFT. Created with BioRender.com.

The aberrant accumulation of Aβ in the brain represents the foremost pathological hallmark of AD. CatB, a cysteine lysosomal protease, facilitates the processing and secretion of IL-1β by microglia. CatB also exhibits secretase activity and participates in the AβPP processing and Aβ generation (Wu et al., 2013; Embury et al., 2017). Chronic systemic exposure to *Pg*-derived LPS promotes CatB-dependent microglial activation and Aβ accumulation in middle-aged mice (Wu et al., 2017). LPS may alter the phagosome proteolytic environment, leading to CatB-dependent Aβ accumulation. Microglia-mediated activation of TLR/NF-κB signaling and neuroinflammation contribute significantly to CatB-mediated Aβ accumulation in neurons (Wu et al., 2017; Zhang J. et al., 2018). Consequently, CatB may represent a crucial link between periodontal pathogens and Aβ deposition in AD pathology (Figure 3).

Aβ is generated not only in brain tissue but also originates peripherally (Leira et al., 2019). Blood circulation-derived Aβ may contribute to AD pathogenesis, and therapeutic agents unable to cross the BBB can also decrease Aβ deposition in brain tissue (Bu et al., 2018). Impaired clearance capacity and accumulation of Aβ in the periphery are essential factors contributing to the brain’s aberrant cerebral deposition of Aβ. Approximately 60% of brain Aβ is removed through transport to the peripheral circulation, with phagocytosis by peripheral monocytes/macrophages serving as the primary route of peripheral Aβ clearance (Condic et al., 2014; Yuede et al., 2016). In addition, chronic systemic infection with *Pg* has been demonstrated to enhance the accumulation of Aβ in peripheral monocytes/macrophages through activation of CatB/NF-κB signaling (Nie et al., 2019). Additionally, chronic *Pg* infection may enhance the transport of Aβ from the periphery into the brain through up-regulation of receptors for advanced glycation end products (RAGE) expression in brain endothelial cells. Significantly, CatB plays a pivotal role in regulating NF-κB/RAGE expression (Zeng et al., 2021).

### 6.2 Mechanisms associated with Tau hyperphosphorylation by periodontal pathogens

Microtubule-associated protein Tau, primarily located in neuronal axons, preserves complex neuronal microarchitecture, including microtubule assembly and stabilizing structures (Tang et al., 2021). Phosphorylation of threonine-proline or serine-proline residues represents a distinctive feature of Tau protein in AD patients (Jiang et al., 2021). Over 40 serine/threonine phosphorylation sites have been identified in AD patients. Hyperphosphorylated Tau has been demonstrated to be associated with synaptic loss, cytoskeletal damage, and dysfunction in axonal transport in AD (Haditsch et al., 2020; Jiang et al., 2021; Tang et al., 2021). Consequently, the sites involved in Tau hyperphosphorylation may mediate AD initiation and progression.

The direct causes of Tau hyperphosphorylation are increased protein phosphokinase activity and/or decreased phosphatase activity. GSK-3β serves as a regulator of microglia-mediated neuroinflammation. The Tau protein phosphorylation stimulated by GSK-3β is susceptible to self-aggregation in a toxic manner. In the mouse models, *Pg*-derived LPS and OMVs trigger the release of inflammatory factors from microglia in the brain, mediated by GSK-3β (Wei et al., 2020; Jiang et al., 2021). The chronic oral infection with *Td* promotes Tau hyperphosphorylation at the sites of Ser396, Thr181, and Thr231 in the mice by elevating GSK-3β activity (Tang et al., 2022). Given association between GSK-3β activation and NF-κB/P65 signaling pathway, it is plausible that periodontal pathogens enhance GSK-3β activity through the activation of the NF-κB pathway (Cao et al., 2017). In turn, it elicits the production of inflammatory factors by microglia, thereby initiating neuroinflammation and associated pathologies.

The protein phosphatase 2A (PP2A) represents a serine–threonine phosphatase in cellular signaling pathways. Rats with *Pg* infection exhibit Tau hyperphosphorylation in the hippocampus, IL-1β up-regulating, and PP2A decreasing, which leads to neuroinflammation in the hippocampus. However, promoting PP2A expression mitigates Tau hyperphosphorylation (Tang et al., 2021). It has been proposed that a reciprocal regulation exists between PP2A and GSK-3β, either through direct or indirect modulation of each other’s activity (Wang et al., 2015). Bacterial-derived brain inflammation may exacerbate Tau pathology through up-regulating GSK-3β activity, while PP2A inhibition decreases GSK-3β activity, promoting phosphorylation at the Ser9 site. Consequently, the PP2A/GSK-3β signaling pathway may mediate the pathological neuroinflammation and Tau hyperphosphorylation induced by periodontal pathogens.

## 7 Conclusion and future prospects

Persuasive evidence indicates that periodontal pathogens contribute to the AD progression by way of the oral-brain axis. Periodontal infections (*Pg*, *Td*, *Fn*, etc.), gingipains, LPS, and OMVs have induced AD-associated neuropathological and behavioral alterations in mouse models, including Aβ plaques, NFT resulting from Tau hyperphosphorylation, extensive neuroinflammation, BBB defects, and impaired cognitive capacity. Periodontal pathogens and/or their toxins could potentially promote Aβ production, activate microglia, release inflammatory factors, and trigger neuronal damage. Persistent neuroinflammation and neurodegeneration may contribute to and further exacerbate AD-related pathology. Notably, *Pg*-derived LPS induced Tau hyperphosphorylation without Aβ deposition, suggesting that Tau hyperphosphorylation could result from systemic exposure to the periodontal pathogen and/or their virulence factors in an Aβ-enriched brain environment (Jiang et al., 2021).

Globally, managing AD poses a significant challenge, encompassing both medical and financial aspects. In contrast, periodontitis is considered interventional or modifiable. Maintaining periodontal health through preventing periodontal disease or targeting key pathogens and/or their virulence factors could potentially benefit the oral-brain axis, influencing the onset and progression of AD. Oral healthcare, particularly the preservation of a healthy and stable oral microbiome, is of paramount importance. Regular oral examinations, oral hygiene management, and periodontal health interventions could potentially reduce the unnecessary AD burden for some individuals. Relevant studies have indicated that Aβ is an antimicrobial peptide, which suggests a potential “antibacterial protection hypothesis” in AD pathology (Soscia et al., 2010; Kumar et al., 2016; Spitzer et al., 2016; Abbott, 2020). Considering the potential role of antibiotics in ameliorating AD conditions, narrow-spectrum antibiotics specifically targeting AD could hold incredible promise, with gingipain as a potential target (Dominy et al., 2019). Moreover, CatB plays a crucial role in neuroinflammation and Aβ deposition, while PP2A induces Tau hyperphosphorylation in neurons through activation of GSK-3β. Inhibition of CatB and PP2A activity could potentially represent a promising therapeutic approach for patients with sporadic AD (Liu et al., 2017; Wu et al., 2017).

The pathogenesis of periodontitis primarily results from oral microbiome dysbiosis, and periodontitis’ influence on AD-related brain immunity and neuroinflammation may extend beyond the influence of a single microorganism. It is likely the consequence of prolonged exposure to multiple organisms and/or associated toxins. Moreover, the research on periodontal pathogens and/or their virulence factors influencing AD-related pathology through the oral-brain axis have primarily been investigated in animal models and cell experiments *in vitro*, with possible variations observed in human subjects. Furthermore, despite a growing body of research on the oral-brain axis in AD pathology, most studies are confined to phenotypic observations. The underlying mechanism of periodontal pathogens’ oral-brain axis remains obscure. The association between periodontal pathogens and AD, as indicated by the oral-brain axis, necessitates further investigation.

## Author contributions

RL: Conceptualization, Writing – original draft, Writing – review & editing. JW: Conceptualization, Writing – original draft, Writing – review & editing. WX: Supervision, Writing – review & editing. YL: Supervision, Writing – review & editing. HF: Writing – review & editing. HZ: Writing – review & editing. YP: Writing – review & editing. YH: Conceptualization, Supervision, Writing – review & editing. QY: Conceptualization, Supervision, Writing – review & editing.

## References

[ref1] Abbott A. (2020). Are infections seeding some cases of Alzheimer's disease? Nature 587, 22–25. doi: 10.1038/d41586-020-03084-9, PMID: 33149296

[ref2] Allen H. B. (2016). Alzheimer's disease: assessing the role of spirochetes, biofilms, the immune system, and amyloid-beta with regard to potential treatment and prevention. J. Alzheimers Dis. 53, 1271–1276. doi: 10.3233/JAD-160388, PMID: 27372648 PMC5008232

[ref3] Allen H. B. (2021). A novel approach to the treatment and prevention of Alzheimer's disease based on the pathology and microbiology. J. Alzheimers Dis. 84, 61–67. doi: 10.3233/JAD-210429 34542071 PMC8609710

[ref4] Alzheimer's Disease International. (2021). World Alzheimer Report 2021. Available at: https://www.alzint.org/resource/world-alzheimer-report-2021/.

[ref5] Aravindraja C. Sakthivel R. Liu X. Goodwin M. Veena P. Godovikova V.. (2022). Intracerebral but not peripheral infection of live *Porphyromonas gingivalis* exacerbates Alzheimer's disease like amyloid pathology in APP-TgCRND8 mice. Int. J. Mol. Sci. 23:3328. doi: 10.3390/ijms23063328, PMID: 35328748 PMC8954230

[ref6] Aruni W. Chioma O. Fletcher H. M. (2014). *Filifactor alocis*: the newly discovered kid on the block with special talents. J. Dent. Res. 93, 725–732. doi: 10.1177/0022034514538283, PMID: 24898946 PMC4126222

[ref7] Aruni A. W. Mishra A. Dou Y. Chioma O. Hamilton B. N. Fletcher H. M. (2015). *Filifactor alocis*--a new emerging periodontal pathogen. Microbes Infect. 17, 517–530. doi: 10.1016/j.micinf.2015.03.011, PMID: 25841800 PMC4485945

[ref8] Bagheri-Mohammadi S. (2021). Microglia in Alzheimer's disease: the role of stem cell-microglia interaction in brain homeostasis. Neurochem. Res. 46, 141–148. doi: 10.1007/s11064-020-03162-4, PMID: 33174075

[ref9] Benedyk M. Marczyk A. Chruscicka B. (2019). Type IX secretion system is pivotal for expression of gingipain-associated virulence of *Porphyromonas gingivalis*. Mol Oral Microbiol 34, 237–244. doi: 10.1111/omi.12268, PMID: 31432617

[ref10] Bu X. L. Xiang Y. Jin W. S. Wang J. Shen L. L. Huang Z. L.. (2018). Blood-derived amyloid-beta protein induces Alzheimer's disease pathologies. Mol. Psychiatry 23, 1948–1956. doi: 10.1038/mp.2017.204, PMID: 29086767

[ref11] Cao Q. Karthikeyan A. Dheen S. T. Kaur C. Ling E. A. (2017). Production of proinflammatory mediators in activated microglia is synergistically regulated by Notch-1, glycogen synthase kinase (GSK-3beta) and NF-kappaB/p65 signalling. PLoS One 12:e0186764. doi: 10.1371/journal.pone.0186764, PMID: 29049420 PMC5648239

[ref12] Carter C. J. France J. Crean S. Singhrao S. K. (2017). The *Porphyromonas gingivalis*/host Interactome shows enrichment in GWASdb genes related to Alzheimer's disease, diabetes and cardiovascular diseases. Front. Aging Neurosci. 9:408. doi: 10.3389/fnagi.2017.00408, PMID: 29311898 PMC5732932

[ref13] Chi L. Cheng X. Lin L. Yang T. Sun J. Feng Y.. (2021). *Porphyromonas gingivalis*-induced cognitive impairment is associated with gut Dysbiosis, Neuroinflammation, and Glymphatic dysfunction. Front. Cell. Infect. Microbiol. 11:755925. doi: 10.3389/fcimb.2021.755925, PMID: 34926316 PMC8672439

[ref14] Cho J. J. Xu Z. Parthasarathy U. Drashansky T. T. Helm E. Y. Zuniga A. N.. (2019). Hectd3 promotes pathogenic Th17 lineage through Stat3 activation and Malt1 signaling in neuroinflammation. Nat. Commun. 10:701. doi: 10.1038/s41467-019-08605-3 30741923 PMC6370850

[ref15] Chou R. C. Kane M. Ghimire S. Gautam S. Gui J. (2016). Treatment for rheumatoid arthritis and risk of Alzheimer's disease: a nested case-control analysis. CNS Drugs 30, 1111–1120. doi: 10.1007/s40263-016-0374-z, PMID: 27470609 PMC5585782

[ref16] Chukkapalli S. Rivera-Kweh M. Gehlot P. Velsko I. Bhattacharyya I. Calise S. J.. (2016). Periodontal bacterial colonization in synovial tissues exacerbates collagen-induced arthritis in B10.RIII mice. Arthritis Res. Ther. 18:161. doi: 10.1186/s13075-016-1056-4, PMID: 27405639 PMC4942913

[ref17] Condic M. Oberstein T. J. Herrmann M. Reimann M. C. Kornhuber J. Maler J. M.. (2014). N-truncation and pyroglutaminylation enhances the opsonizing capacity of Abeta-peptides and facilitates phagocytosis by macrophages and microglia. Brain Behav. Immun. 41, 116–125. doi: 10.1016/j.bbi.2014.05.003, PMID: 24876064

[ref18] Coureuil M. Lecuyer H. Bourdoulous S. Nassif X. (2017). A journey into the brain: insight into how bacterial pathogens cross blood-brain barriers. Nat. Rev. Microbiol. 15, 149–159. doi: 10.1038/nrmicro.2016.178, PMID: 28090076

[ref19] Darveau R. P. Pham T. T. Lemley K. Reife R. A. Bainbridge B. W. Coats S. R.. (2004). *Porphyromonas gingivalis* lipopolysaccharide contains multiple lipid a species that functionally interact with both toll-like receptors 2 and 4. Infect. Immun. 72, 5041–5051. doi: 10.1128/IAI.72.9.5041-5051.2004, PMID: 15321997 PMC517442

[ref20] das S. Ansel K. M. Bitzer M. Breakefield X. O. Charest A. Galas D. J.. (2019). The extracellular RNA communication consortium: establishing foundational knowledge and Technologies for Extracellular RNA research. Cell 177, 231–242. doi: 10.1016/j.cell.2019.03.023, PMID: 30951667 PMC6601620

[ref21] Díaz-Zúñiga J. More J. Melgar-Rodríguez S. Jiménez-Unión M. Villalobos-Orchard F. Muñoz-Manríquez C.. (2020). Alzheimer's disease-like pathology triggered by *Porphyromonas gingivalis* in wild type rats is serotype dependent. Front. Immunol. 11:588036. doi: 10.3389/fimmu.2020.588036, PMID: 33240277 PMC7680957

[ref22] Díaz-Zúñiga J. Muñoz Y. Melgar-Rodríguez S. More J. Bruna B. Lobos P.. (2019). Serotype b of *Aggregatibacter actinomycetemcomitans* triggers pro-inflammatory responses and amyloid beta secretion in hippocampal cells: a novel link between periodontitis and Alzheimer’s disease? J. Oral Microbiol. 11:1586423. doi: 10.1080/20002297.2019.1586423, PMID: 31044031 PMC6484476

[ref23] Ding Y. Ren J. Yu H. Yu W. Zhou Y. (2018). *Porphyromonas gingivalis*, a periodontitis causing bacterium, induces memory impairment and age-dependent neuroinflammation in mice. Immun. Ageing 15:6. doi: 10.1186/s12979-017-0110-7, PMID: 29422938 PMC5791180

[ref24] Dominy S. S. Lynch C. Ermini F. Benedyk M. Marczyk A. Konradi A.. (2019). *Porphyromonas gingivalis* in Alzheimer's disease brains: evidence for disease causation and treatment with small-molecule inhibitors. Sci. Adv. 5:eaau3333. doi: 10.1126/sciadv.aau3333, PMID: 30746447 PMC6357742

[ref25] Dorszewska J. Prendecki M. Oczkowska A. Dezor M. Kozubski W. (2016). Molecular basis of familial and sporadic Alzheimer's disease. Curr. Alzheimer Res. 13, 952–963. doi: 10.2174/1567205013666160314150501, PMID: 26971934

[ref26] Embury C. M. Dyavarshetty B. Lu Y. Wiederin J. L. Ciborowski P. Gendelman H. E.. (2017). Cathepsin B improves ss-amyloidosis and learning and memory in models of Alzheimer's disease. J. Neuroimmune Pharmacol. 12, 340–352. doi: 10.1007/s11481-016-9721-6, PMID: 27966067 PMC5405105

[ref27] Farrugia C. Stafford G. P. Murdoch C. (2020). *Porphyromonas gingivalis* outer membrane vesicles increase vascular permeability. J. Dent. Res. 99, 1494–1501. doi: 10.1177/0022034520943187, PMID: 32726180 PMC7684789

[ref28] Fleetwood A. J. Lee M. K. S. Singleton W. Achuthan A. Lee M. C. O'Brien-Simpson N. M.. (2017). Metabolic remodeling, Inflammasome activation, and Pyroptosis in macrophages stimulated by *Porphyromonas gingivalis* and its outer membrane vesicles. Front. Cell. Infect. Microbiol. 7:351. doi: 10.3389/fcimb.2017.00351, PMID: 28824884 PMC5543041

[ref29] Fonseca M. I. Chu S. H. Hernandez M. X. Fang M. J. Modarresi L. Selvan P.. (2017). Cell-specific deletion of C1qa identifies microglia as the dominant source of C1q in mouse brain. J. Neuroinflammation 14:48. doi: 10.1186/s12974-017-0814-9, PMID: 28264694 PMC5340039

[ref30] Fonseca M. I. Zhou J. Botto M. Tenner A. J. (2004). Absence of C1q leads to less neuropathology in transgenic mouse models of Alzheimer's disease. J. Neurosci. 24, 6457–6465. doi: 10.1523/JNEUROSCI.0901-04.2004, PMID: 15269255 PMC6729885

[ref31] Foschi F. Izard J. Sasaki H. Sambri V. Prati C. Müller R.. (2006). *Treponema denticola* in disseminating endodontic infections. J. Dent. Res. 85, 761–765. doi: 10.1177/154405910608500814, PMID: 16861296 PMC3579618

[ref32] Gil-Montoya J. A. Sanchez-Lara I. Carnero-Pardo C. Fornieles F. Montes J. Vilchez R.. (2015). Is periodontitis a risk factor for cognitive impairment and dementia? A case-control study. J. Periodontol 86, 244–253. doi: 10.1902/jop.2014.140340 25345338

[ref33] Gong T. Chen Q. Mao H. Zhang Y. Ren H. Xu M.. (2022). Outer membrane vesicles of *Porphyromonas gingivalis* trigger NLRP3 inflammasome and induce neuroinflammation, tau phosphorylation, and memory dysfunction in mice. Front. Cell. Infect. Microbiol. 12:925435. doi: 10.3389/fcimb.2022.925435, PMID: 36017373 PMC9397999

[ref34] Gong Y. Liu Z. Zhou P. Li J. Miao Y.-B. (2023). Biomimetic nanocarriers harnessing microbial metabolites usher the path for brain disease therapy. Nano TransMed 2:100020. doi: 10.1016/j.ntm.2023.100020

[ref35] Gu Y. Wu Z. Zeng F. Jiang M. Teeling J. L. Ni J.. (2020). Systemic exposure to lipopolysaccharide from *Porphyromonas gingivalis* induces bone loss-correlated Alzheimer's disease-like pathologies in middle-aged mice. J. Alzheimers Dis. 78, 61–74. doi: 10.3233/JAD-200689, PMID: 32925065

[ref36] Guan G. Polonowita A. Sun Q. Mei L. (2023). Immune-mediated conditions and cellular biomarkers for early diagnosis of oral diseases. Nano TransMed 2:100001. doi: 10.1016/j.ntm.2023.100001

[ref37] Guo H. Li B. Yao H. Liu D. Chen R. Zhou S.. (2023). Profiling the oral microbiomes in patients with Alzheimer's disease. Oral Dis. 29, 1341–1355. doi: 10.1111/odi.14110, PMID: 34931394

[ref38] Ha J. Y. Choi S. Y. Lee J. H. Hong S. H. Lee H. J. (2020). Delivery of Periodontopathogenic extracellular vesicles to brain monocytes and microglial IL-6 promotion by RNA cargo. Front. Mol. Biosci. 7:596366. doi: 10.3389/fmolb.2020.596366, PMID: 33330627 PMC7732644

[ref39] Haditsch U. Roth T. Rodriguez L. Hancock S. Cecere T. Nguyen M.. (2020). Alzheimer's disease-like neurodegeneration in *Porphyromonas gingivalis* infected neurons with persistent expression of active Gingipains. J. Alzheimers Dis. 75, 1361–1376. doi: 10.3233/JAD-200393, PMID: 32390638 PMC7369049

[ref40] Han E. C. Choi S. Y. Lee Y. Park J. W. Hong S. H. Lee H. J. (2019). Extracellular RNAs in periodontopathogenic outer membrane vesicles promote TNF-alpha production in human macrophages and cross the blood-brain barrier in mice. FASEB J. 33, 13412–13422. doi: 10.1096/fj.201901575R, PMID: 31545910 PMC6894046

[ref41] Hao X. Li Z. Li W. Katz J. Michalek S. M. Barnum S. R.. (2022). Periodontal infection aggravates C1q-mediated microglial activation and synapse pruning in Alzheimer's mice. Front. Immunol. 13:816640. doi: 10.3389/fimmu.2022.816640, PMID: 35178049 PMC8845011

[ref42] Hodson R. (2018). Alzheimer's disease. Nature 559:S1. doi: 10.1038/d41586-018-05717-6 30046078

[ref43] Holmer J. Aho V. Eriksdotter M. Paulin L. Pietiainen M. Auvinen P.. (2021). Subgingival microbiota in a population with and without cognitive dysfunction. J. Oral Microbiol. 13:1854552. doi: 10.1080/20002297.2020.1854552, PMID: 33537116 PMC7833025

[ref44] Hong S. Beja-Glasser V. F. Nfonoyim B. M. Frouin A. Li S. Ramakrishnan S.. (2016). Complement and microglia mediate early synapse loss in Alzheimer mouse models. Science 352, 712–716. doi: 10.1126/science.aad8373, PMID: 27033548 PMC5094372

[ref45] Huang J. K. Ma P. L. Ji S. Y. Zhao X. L. Tan J. X. Sun X. J.. (2013). Age-dependent alterations in the presynaptic active zone in a Drosophila model of Alzheimer's disease. Neurobiol. Dis. 51, 161–167. doi: 10.1016/j.nbd.2012.11.006 23149068

[ref46] Huang W. Zeng F. Gu Y. Jiang M. Zhang X. Yan X.. (2021). *Porphyromonas Gingivalis* infection induces synaptic failure via increased IL-1beta production in leptomeningeal cells. J. Alzheimers Dis. 83, 665–681. doi: 10.3233/JAD-210031, PMID: 34334391

[ref47] Ide M. Harris M. Stevens A. Sussams R. Hopkins V. Culliford D.. (2016). Periodontitis and cognitive decline in Alzheimer's disease. PLoS One 11:e0151081. doi: 10.1371/journal.pone.0151081, PMID: 26963387 PMC4786266

[ref48] Ilievski V. Zuchowska P. K. Green S. J. Toth P. T. Ragozzino M. E. le K.. (2018). Chronic oral application of a periodontal pathogen results in brain inflammation, neurodegeneration and amyloid beta production in wild type mice. PLoS One 13:e0204941. doi: 10.1371/journal.pone.0204941, PMID: 30281647 PMC6169940

[ref49] Italiani P. Puxeddu I. Napoletano S. Scala E. Melillo D. Manocchio S.. (2018). Circulating levels of IL-1 family cytokines and receptors in Alzheimer's disease: new markers of disease progression? J. Neuroinflammation 15:342. doi: 10.1186/s12974-018-1376-1, PMID: 30541566 PMC6292179

[ref50] Iwasaki M. Kimura Y. Ogawa H. Yamaga T. Ansai T. Wada T.. (2019). Periodontitis, periodontal inflammation, and mild cognitive impairment: a 5-year cohort study. J. Periodontal Res. 54, 233–240. doi: 10.1111/jre.12623, PMID: 30345659

[ref51] Jain S. Darveau R. P. (2010). Contribution of *Porphyromonas gingivalis* lipopolysaccharide to periodontitis. Periodontol. 54, 53–70. doi: 10.1111/j.1600-0757.2009.00333.x PMC2943730 20712633

[ref52] Jia L. Du Y. Chu L. Zhang Z. Li F. Lyu D.. (2020). Prevalence, risk factors, and management of dementia and mild cognitive impairment in adults aged 60 years or older in China: a cross-sectional study. Lancet Public Health 5, e661–e671. doi: 10.1016/S2468-2667(20)30185-7 33271079

[ref53] Jiang M. Zhang X. Yan X. Mizutani S. Kashiwazaki H. Ni J.. (2021). GSK3beta is involved in promoting Alzheimer's disease pathologies following chronic systemic exposure to *Porphyromonas gingivalis* lipopolysaccharide in amyloid precursor protein(NL-F/NL-F) knock-in mice. Brain Behav. Immun. 98, 1–12. doi: 10.1016/j.bbi.2021.08.213, PMID: 34391814 PMC8849844

[ref54] Kirkley K. S. Popichak K. A. Afzali M. F. Legare M. E. Tjalkens R. B. (2017). Microglia amplify inflammatory activation of astrocytes in manganese neurotoxicity. J. Neuroinflammation 14:99. doi: 10.1186/s12974-017-0871-0, PMID: 28476157 PMC5418760

[ref55] Kitazawa M. Oddo S. Yamasaki T. R. Green K. N. LaFerla F. M. (2005). Lipopolysaccharide-induced inflammation exacerbates tau pathology by a cyclin-dependent kinase 5-mediated pathway in a transgenic model of Alzheimer's disease. J. Neurosci. 25, 8843–8853. doi: 10.1523/JNEUROSCI.2868-05.2005, PMID: 16192374 PMC6725603

[ref56] Kumar D. K. Choi S. H. Washicosky K. J. Eimer W. A. Tucker S. Ghofrani J.. (2016). Amyloid-beta peptide protects against microbial infection in mouse and worm models of Alzheimer's disease. Sci. Transl. Med. 8:340ra72. doi: 10.1126/scitranslmed.aaf1059, PMID: 27225182 PMC5505565

[ref57] Lei S. Li J. Yu J. Li F. Pan Y. Chen X.. (2023). *Porphyromonas gingivalis* bacteremia increases the permeability of the blood-brain barrier via the Mfsd2a/Caveolin-1 mediated transcytosis pathway. Int. J. Oral Sci. 15:3. doi: 10.1038/s41368-022-00215-y, PMID: 36631446 PMC9834243

[ref58] Leira Y. Iglesias-Rey R. Gomez-Lado N. Aguiar P. Campos F. D'Aiuto F.. (2019). *Porphyromonas gingivalis* lipopolysaccharide-induced periodontitis and serum amyloid-beta peptides. Arch. Oral Biol. 99, 120–125. doi: 10.1016/j.archoralbio.2019.01.008, PMID: 30665148

[ref59] Lewis K. M. Bharadwaj U. Eckols T. K. Kolosov M. Kasembeli M. M. Fridley C.. (2015). Small-molecule targeting of signal transducer and activator of transcription (STAT) 3 to treat non-small cell lung cancer. Lung Cancer 90, 182–190. doi: 10.1016/j.lungcan.2015.09.014, PMID: 26410177 PMC4619129

[ref60] Liu Y. Wu Z. Nakanishi Y. Ni J. Hayashi Y. Takayama F.. (2017). Infection of microglia with *Porphyromonas gingivalis* promotes cell migration and an inflammatory response through the gingipain-mediated activation of protease-activated receptor-2 in mice. Sci. Rep. 7:11759. doi: 10.1038/s41598-017-12173-1, PMID: 28924232 PMC5603557

[ref61] Lu Z. He R. Zhang Y. Li B. Li F. Fu Y.. (2023). Relationship between whole-blood magnesium and cognitive performance among Chinese adults. Nutrients 15:2706. doi: 10.3390/nu15122706, PMID: 37375610 PMC10304450

[ref62] Ma X. Shin Y. J. Yoo J. W. Park H. S. Kim D. H. (2023). Extracellular vesicles derived from *Porphyromonas gingivalis* induce vagus nerve-mediated cognitive impairment. J. Adv. Res. 54, 293–303. doi: 10.1016/j.jare.2023.02.006, PMID: 36796586 PMC10703712

[ref63] Miklossy J. (1993). Alzheimerʼs disease—a spirochetosis? Neuroreport 4, 841–848. doi: 10.1097/00001756-199307000-00002, PMID: 8369471

[ref64] Miklossy J. (2011). Alzheimer's disease - a neurospirochetosis. Analysis of the evidence following Koch's and Hill's criteria. J. Neuroinflammation 8:90. doi: 10.1186/1742-2094-8-90, PMID: 21816039 PMC3171359

[ref65] Nakayama K. (2015). *Porphyromonas gingivalis* and related bacteria: from colonial pigmentation to the type IX secretion system and gliding motility. J. Periodontal Res. 50, 1–8. doi: 10.1111/jre.12255, PMID: 25546073 PMC4674972

[ref66] Nation D. A. Sweeney M. D. Montagne A. Sagare A. P. D'Orazio L. M. Pachicano M.. (2019). Blood-brain barrier breakdown is an early biomarker of human cognitive dysfunction. Nat. Med. 25, 270–276. doi: 10.1038/s41591-018-0297-y, PMID: 30643288 PMC6367058

[ref67] Nie R. Wu Z. Ni J. Zeng F. Yu W. Zhang Y.. (2019). *Porphyromonas gingivalis* infection induces amyloid-beta accumulation in monocytes/macrophages. J. Alzheimers Dis. 72, 479–494. doi: 10.3233/JAD-190298, PMID: 31594220

[ref68] Noble J. M. Borrell L. N. Papapanou P. N. Elkind M. S. Scarmeas N. Wright C. B. (2009). Periodontitis is associated with cognitive impairment among older adults: analysis of NHANES-III. J. Neurol. Neurosurg. Psychiatry 80, 1206–1211. doi: 10.1136/jnnp.2009.174029, PMID: 19419981 PMC3073380

[ref69] Noble J. M. Scarmeas N. Celenti R. S. Elkind M. S. Wright C. B. Schupf N.. (2014). Serum IgG antibody levels to periodontal microbiota are associated with incident Alzheimer disease. PLoS One 9:e114959. doi: 10.1371/journal.pone.0114959, PMID: 25522313 PMC4270775

[ref70] Nonaka S. Kadowaki T. Nakanishi H. (2022). Secreted gingipains from *Porphyromonas gingivalis* increase permeability in human cerebral microvascular endothelial cells through intracellular degradation of tight junction proteins. Neurochem. Int. 154:105282. doi: 10.1016/j.neuint.2022.105282, PMID: 35032577

[ref71] Oberstein T. J. Taha L. Spitzer P. Hellstern J. Herrmann M. Kornhuber J.. (2018). Imbalance of circulating T(h)17 and regulatory T cells in Alzheimer's disease: a case control study. Front. Immunol. 9:1213. doi: 10.3389/fimmu.2018.01213, PMID: 29915582 PMC5994416

[ref72] Olsen I. Singhrao S. K. (2015). Can oral infection be a risk factor for Alzheimer's disease? J. Oral Microbiol. 7:29143. doi: 10.3402/jom.v7.29143, PMID: 26385886 PMC4575419

[ref73] Olsen I. Yilmaz O. (2016). Modulation of inflammasome activity by *Porphyromonas gingivalis* in periodontitis and associated systemic diseases. J. Oral Microbiol. 8:30385. doi: 10.3402/jom.v8.30385, PMID: 26850450 PMC4744328

[ref74] Panzarella V. Mauceri R. Baschi R. Maniscalco L. Campisi G. Monastero R. (2022). Oral health status in subjects with amnestic mild cognitive impairment and Alzheimer's disease: data from the Zabut aging project. J. Alzheimers Dis. 87, 173–183. doi: 10.3233/JAD-200385, PMID: 32508326 PMC9277678

[ref75] Park J. Wetzel I. Marriott I. Dréau D. D’Avanzo C. Kim D. Y.. (2018). A 3D human triculture system modeling neurodegeneration and neuroinflammation in Alzheimer's disease. Nat. Neurosci. 21, 941–951. doi: 10.1038/s41593-018-0175-4, PMID: 29950669 PMC6800152

[ref76] Poole S. Singhrao S. K. Kesavalu L. Curtis M. A. Crean S. (2013). Determining the presence of periodontopathic virulence factors in short-term postmortem Alzheimer's disease brain tissue. J. Alzheimers Dis. 36, 665–677. doi: 10.3233/JAD-121918, PMID: 23666172

[ref77] Pritchard A. B. Fabian Z. Lawrence C. L. Morton G. Crean S. Alder J. E. (2022). An investigation into the effects of outer membrane vesicles and lipopolysaccharide of *Porphyromonas gingivalis* on blood-brain barrier integrity, permeability, and disruption of scaffolding proteins in a human *in vitro* model. J. Alzheimers Dis. 86, 343–364. doi: 10.3233/JAD-215054, PMID: 35034897

[ref78] Qian X. Zhang S. Duan L. Yang F. Zhang K. Yan F.. (2021). Periodontitis deteriorates cognitive function and impairs neurons and glia in a mouse model of Alzheimer's disease. J. Alzheimers Dis. 79, 1785–1800. doi: 10.3233/JAD-201007, PMID: 33459718

[ref79] Qiu C. Yuan Z. He Z. Chen H. Liao Y. Li S.. (2021). Lipopolysaccharide preparation derived from *Porphyromonas gingivalis* induces a weaker Immuno-inflammatory response in BV-2 microglial cells than *Escherichia coli* by differentially activating TLR2/4-mediated NF-kappaB/STAT3 signaling pathways. Front. Cell. Infect. Microbiol. 11:606986. doi: 10.3389/fcimb.2021.606986, PMID: 33816329 PMC8012810

[ref80] Riviere G. R. Riviere K. H. Smith K. S. (2002). Molecular and immunological evidence of oral Treponema in the human brain and their association with Alzheimer's disease. Oral Microbiol. Immunol. 17, 113–118. doi: 10.1046/j.0902-0055.2001.00100.x 11929559

[ref81] Saffari P. M. Alijanpour S. Takzaree N. Sahebgharani M. Etemad-Moghadam S. Noorbakhsh F.. (2020). Metformin loaded phosphatidylserine nanoliposomes improve memory deficit and reduce neuroinflammation in streptozotocin-induced Alzheimer's disease model. Life Sci. 255:117861. doi: 10.1016/j.lfs.2020.117861, PMID: 32473247

[ref82] Salminen A. Kaarniranta K. Kauppinen A. (2020). ER stress activates immunosuppressive network: implications for aging and Alzheimer's disease. J. Mol. Med. (Berl) 98, 633–650. doi: 10.1007/s00109-020-01904-z, PMID: 32279085 PMC7220864

[ref83] Salter M. W. Beggs S. (2014). Sublime microglia: expanding roles for the guardians of the CNS. Cell 158, 15–24. doi: 10.1016/j.cell.2014.06.008, PMID: 24995975

[ref84] Sansores-Espana D. Carrillo-Avila A. Melgar-Rodriguez S. Diaz-Zuniga J. Martinez-Aguilar V. (2021). Periodontitis and Alzheimer's disease. Med. Oral Patol. Oral Cir. Bucal. 26, e43–e48. doi: 10.4317/medoral.23940, PMID: 32701930 PMC7806353

[ref85] Sansores-España L. D. Melgar-Rodríguez S. Olivares-Sagredo K. Cafferata E. A. Martínez-Aguilar V. M. Vernal R.. (2021). Oral-gut-brain Axis in experimental models of periodontitis: associating gut Dysbiosis with neurodegenerative diseases. Front Aging 2:781582. doi: 10.3389/fragi.2021.781582, PMID: 35822001 PMC9261337

[ref86] Saresella M. La Rosa F. Piancone F. Zoppis M. Marventano I. Calabrese E.. (2016). The NLRP3 and NLRP1 inflammasomes are activated in Alzheimer's disease. Mol. Neurodegener. 11:23. doi: 10.1186/s13024-016-0088-1 26939933 PMC4778358

[ref87] Sato N. Matsumoto T. Kawaguchi S. Seya K. Matsumiya T. Ding J.. (2022). *Porphyromonas gingivalis* lipopolysaccharide induces interleukin-6 and c-c motif chemokine ligand 2 expression in cultured hCMEC/D3 human brain microvascular endothelial cells. Gerodontology 39, 139–147. doi: 10.1111/ger.12545, PMID: 33599317

[ref88] Schmidt T. S. Hayward M. R. Coelho L. P. Li S. S. Costea P. I. Voigt A. Y.. (2019). Extensive transmission of microbes along the gastrointestinal tract. elife 8:e42693. doi: 10.7554/eLife.42693, PMID: 30747106 PMC6424576

[ref89] Sczepanik F. S. C. Grossi M. L. Casati M. Goldberg M. Glogauer M. Fine N.. (2020). Periodontitis is an inflammatory disease of oxidative stress: we should treat it that way. Periodontol. 84, 45–68. doi: 10.1111/prd.12342, PMID: 32844417

[ref90] Shaik-Dasthagirisaheb Y. B. Huang N. Weinberg E. O. Shen S. S. Genco C. A. Gibson F. C. 3rd (2015). Aging and contribution of MyD88 and TRIF to expression of TLR pathway-associated genes following stimulation with *Porphyromonas gingivalis*. J. Periodontal Res. 50, 89–102. doi: 10.1111/jre.12185, PMID: 24862405 PMC4242805

[ref91] Shin H. S. Shin M. S. Ahn Y. B. Choi B. Y. Nam J. H. Kim H. D. (2016). Periodontitis is associated with cognitive impairment in elderly Koreans: results from the Yangpyeong cohort study. J. Am. Geriatr. Soc. 64, 162–167. doi: 10.1111/jgs.13781, PMID: 26782867

[ref92] Singhrao S. K. Harding A. Poole S. Kesavalu L. Crean S. (2015). *Porphyromonas gingivalis* periodontal infection and its putative links with Alzheimer's disease. Mediat. Inflamm. 2015:137357. doi: 10.1155/2015/137357, PMID: 26063967 PMC4430664

[ref93] Soscia S. J. Kirby J. E. Washicosky K. J. Tucker S. M. Ingelsson M. Hyman B.. (2010). The Alzheimer's disease-associated amyloid beta-protein is an antimicrobial peptide. PLoS One 5:e9505. doi: 10.1371/journal.pone.0009505, PMID: 20209079 PMC2831066

[ref94] Sparks Stein P. Steffen M. J. Smith C. Jicha G. Ebersole J. L. Abner E.. (2012). Serum antibodies to periodontal pathogens are a risk factor for Alzheimer's disease. Alzheimers Dement. 8, 196–203. doi: 10.1016/j.jalz.2011.04.006, PMID: 22546352 PMC3712346

[ref95] Spitzer P. Condic M. Herrmann M. Oberstein T. J. Scharin-Mehlmann M. Gilbert D. F.. (2016). Amyloidogenic amyloid-beta-peptide variants induce microbial agglutination and exert antimicrobial activity. Sci. Rep. 6:32228. doi: 10.1038/srep32228, PMID: 27624303 PMC5021948

[ref96] Su X. Tang Z. Lu Z. Liu Y. He W. Jiang J.. (2021). Oral *Treponema denticola* infection induces Abeta (1-40) and Abeta (1-42) accumulation in the Hippocampus of C57BL/6 mice. J. Mol. Neurosci. 71, 1506–1514. doi: 10.1007/s12031-021-01827-5, PMID: 33763842

[ref97] Sung C. E. Huang R. Y. Cheng W. C. Kao T. W. Chen W. L. (2019). Association between periodontitis and cognitive impairment: analysis of national health and nutrition examination survey (NHANES) III. J. Clin. Periodontol. 46, 790–798. doi: 10.1111/jcpe.13155, PMID: 31152592

[ref98] Taati Moghadam M. Amirmozafari N. Mojtahedi A. Bakhshayesh B. Shariati A. Masjedian Jazi F. (2022). Association of perturbation of oral bacterial with incident of Alzheimer's disease: a pilot study. J. Clin. Lab. Anal. 36:e24483. doi: 10.1002/jcla.24483, PMID: 35689551 PMC9279996

[ref99] Takayama F. Hayashi Y. Wu Z. Liu Y. Nakanishi H. (2016). Diurnal dynamic behavior of microglia in response to infected bacteria through the UDP-P2Y6 receptor system. Sci. Rep. 6:30006. doi: 10.1038/srep30006, PMID: 27445174 PMC4956748

[ref100] Tang Z. Cheng X. Su X. Wu L. Cai Q. Wu H. (2022). *Treponema denticola* induces Alzheimer-like tau hyperphosphorylation by activating hippocampal Neuroinflammation in mice. J. Dent. Res. 101, 992–1001. doi: 10.1177/00220345221076772, PMID: 35193423

[ref101] Tang Z. Liang D. Cheng M. Su X. Liu R. Zhang Y.. (2021). Effects of *Porphyromonas gingivalis* and its underlying mechanisms on Alzheimer-like tau hyperphosphorylation in Sprague-Dawley rats. J. Mol. Neurosci. 71, 89–100. doi: 10.1007/s12031-020-01629-1, PMID: 32557144

[ref102] Tetz G. Pinho M. Pritzkow S. Mendez N. Soto C. Tetz V. (2020). Bacterial DNA promotes tau aggregation. Sci. Rep. 10:2369. doi: 10.1038/s41598-020-59364-x, PMID: 32047247 PMC7012890

[ref103] Tetz G. Tetz V. (2021). Bacterial extracellular DNA promotes beta-amyloid aggregation. Microorganisms 9:1301. doi: 10.3390/microorganisms9061301, PMID: 34203755 PMC8232312

[ref104] Tran V. T. A. Kang Y. J. Kim H. K. Kim H. R. Cho H. (2021). Oral pathogenic Bacteria-inducing neurodegenerative microgliosis in human neural cell platform. Int. J. Mol. Sci. 22:6925. doi: 10.3390/ijms22136925, PMID: 34203256 PMC8269080

[ref105] Vernal R. Diaz-Guerra E. Silva A. Sanz M. Garcia-Sanz J. A. (2014a). Distinct human T-lymphocyte responses triggered by *Porphyromonas gingivalis* capsular serotypes. J. Clin. Periodontol. 41, 19–30. doi: 10.1111/jcpe.12176, PMID: 24117627

[ref106] Vernal R. Diaz-Zuniga J. Melgar-Rodriguez S. Pujol M. Diaz-Guerra E. Silva A.. (2014b). Activation of RANKL-induced osteoclasts and memory T lymphocytes by *Porphyromonas gingivalis* is serotype dependant. J. Clin. Periodontol. 41, 451–459. doi: 10.1111/jcpe.12236 24476556

[ref107] Wang R. P. Huang J. Chan K. W. Y. Leung W. K. Goto T. Ho Y. S.. (2023). IL-1beta and TNF-alpha play an important role in modulating the risk of periodontitis and Alzheimer's disease. J. Neuroinflammation 20:71. doi: 10.1186/s12974-023-02747-4, PMID: 36915108 PMC10012546

[ref108] Wang Y. Yang R. Gu J. Yin X. Jin N. Xie S.. (2015). Cross talk between PI3K-AKT-GSK-3beta and PP2A pathways determines tau hyperphosphorylation. Neurobiol. Aging 36, 188–200. doi: 10.1016/j.neurobiolaging.2014.07.035, PMID: 25219467

[ref109] Wei S. Peng W. Mai Y. Li K. Wei W. Hu L.. (2020). Outer membrane vesicles enhance tau phosphorylation and contribute to cognitive impairment. J. Cell. Physiol. 235, 4843–4855. doi: 10.1002/jcp.29362, PMID: 31663118

[ref110] Whelan C. D. Mattsson N. Nagle M. W. Vijayaraghavan S. Hyde C. Janelidze S.. (2019). Multiplex proteomics identifies novel CSF and plasma biomarkers of early Alzheimer's disease. Acta Neuropathol. Commun. 7:169. doi: 10.1186/s40478-019-0795-2, PMID: 31694701 PMC6836495

[ref111] Wu Z. Ni J. Liu Y. Teeling J. L. Takayama F. Collcutt A.. (2017). Cathepsin B plays a critical role in inducing Alzheimer's disease-like phenotypes following chronic systemic exposure to lipopolysaccharide from *Porphyromonas gingivalis* in mice. Brain Behav. Immun. 65, 350–361. doi: 10.1016/j.bbi.2017.06.002, PMID: 28610747

[ref112] Wu H. Qiu W. Zhu X. Li X. Xie Z. Carreras I.. (2022). The periodontal pathogen *Fusobacterium nucleatum* exacerbates Alzheimer's pathogenesis via specific pathways. Front. Aging Neurosci. 14:912709. doi: 10.3389/fnagi.2022.912709, PMID: 35813949 PMC9260256

[ref113] Wu L. Su X. Tang Z. Jian L. Zhu H. Cheng X.. (2022). Treponema denticola induces neuronal apoptosis by promoting amyloid-beta accumulation in mice. Pathogens 11. doi: 10.3390/pathogens11101150, PMID: 36297207 PMC9610539

[ref114] Wu Z. Sun L. Hashioka S. Yu S. Schwab C. Okada R.. (2013). Differential pathways for interleukin-1beta production activated by chromogranin a and amyloid beta in microglia. Neurobiol. Aging 34, 2715–2725. doi: 10.1016/j.neurobiolaging.2013.05.018 23831373

[ref115] Yamada C. Akkaoui J. Ho A. Duarte C. Deth R. Kawai T.. (2020). Potential role of Phosphoglycerol Dihydroceramide produced by periodontal pathogen *Porphyromonas gingivalis* in the pathogenesis of Alzheimer's disease. Front. Immunol. 11:591571. doi: 10.3389/fimmu.2020.591571, PMID: 33329577 PMC7719741

[ref116] Yamatake K. Maeda M. Kadowaki T. Takii R. Tsukuba T. Ueno T.. (2007). Role for gingipains in *Porphyromonas gingivalis* traffic to phagolysosomes and survival in human aortic endothelial cells. Infect. Immun. 75, 2090–2100. doi: 10.1128/IAI.01013-06, PMID: 17296756 PMC1865784

[ref117] Yan C. Diao Q. Zhao Y. Zhang C. He X. Huang R.. (2022). *Fusobacterium nucleatum* infection-induced neurodegeneration and abnormal gut microbiota composition in Alzheimer's disease-like rats. Front. Neurosci. 16:884543. doi: 10.3389/fnins.2022.884543, PMID: 36188448 PMC9523129

[ref118] Yang I. Arthur R. A. Zhao L. Clark J. Hu Y. Corwin E. J.. (2021). The oral microbiome and inflammation in mild cognitive impairment. Exp. Gerontol. 147:111273. doi: 10.1016/j.exger.2021.111273 33556534

[ref119] Yuede C. M. Lee H. Restivo J. L. Davis T. A. Hettinger J. C. Wallace C. E.. (2016). Rapid in vivo measurement of beta-amyloid reveals biphasic clearance kinetics in an Alzheimer's mouse model. J. Exp. Med. 213, 677–685. doi: 10.1084/jem.20151428, PMID: 27069115 PMC4854730

[ref120] Zeng F. Liu Y. Huang W. Qing H. Kadowaki T. Kashiwazaki H.. (2021). Receptor for advanced glycation end products up-regulation in cerebral endothelial cells mediates cerebrovascular-related amyloid beta accumulation after *Porphyromonas gingivalis* infection. J. Neurochem. 158, 724–736. doi: 10.1111/jnc.15096, PMID: 32441775 PMC8451939

[ref121] Zhang Y. Feng S. Nie K. Li Y. Gao Y. Gan R.. (2018). TREM2 modulates microglia phenotypes in the neuroinflammation of Parkinson's disease. Biochem. Biophys. Res. Commun. 499, 797–802. doi: 10.1016/j.bbrc.2018.03.226, PMID: 29621548

[ref122] Zhang L. Gao L. Xu C. Li X. Wang P. Zhang C.. (2019). *Porphyromonas gingivalis* lipopolysaccharide promotes T-helper 17 cell differentiation from human CD4(+) naive T cells via toll-like receptor-2 in vitro. Arch. Oral Biol. 107:104483. doi: 10.1016/j.archoralbio.2019.104483, PMID: 31351339

[ref123] Zhang J. Yu C. Zhang X. Chen H. Dong J. Lu W.. (2018). *Porphyromonas gingivalis* lipopolysaccharide induces cognitive dysfunction, mediated by neuronal inflammation via activation of the TLR4 signaling pathway in C57BL/6 mice. J. Neuroinflammation 15:37. doi: 10.1186/s12974-017-1052-x 29426327 PMC5810193

[ref124] Zhang X. Zhang X. Qiu C. Shen H. Zhang H. He Z.. (2021). The imbalance of Th17/Treg via STAT3 activation modulates cognitive impairment in *P. gingivalis* LPS-induced periodontitis mice. J. Leukoc. Biol. 110, 511–524. doi: 10.1002/JLB.3MA0521-742RRR, PMID: 34342041

